# Tunneling induced absorption with competing Nonlinearities

**DOI:** 10.1038/srep38251

**Published:** 2016-12-13

**Authors:** Yandong Peng, Aihong Yang, Yan Xu, Peng Wang, Yang Yu, Hongju Guo, Tingqi Ren

**Affiliations:** 1 State Key Laboratory of Mining Disaster Prevention and Control Co-founded by Shandong Province and the Ministry of Science and Technology, Shandong University of Science and Technology, Qingdao 266590, China; 2 Physics Research Laboratory, Shanghai Publishing and Printing College, University of Shanghai for Science and Technology, Shanghai, 200093, China

## Abstract

We investigate tunneling induced nonlinear absorption phenomena in a coupled quantum-dot system. Resonant tunneling causes constructive interference in the nonlinear absorption that leads to an increase of more than an order of magnitude over the maximum absorption in a coupled quantum dot system without tunneling. Resonant tunneling also leads to a narrowing of the linewidth of the absorption peak to a sublinewidth level. Analytical expressions show that the enhanced nonlinear absorption is largely due to the fifth-order nonlinear term. Competition between third- and fifth-order nonlinearities leads to an anomalous dispersion of the total susceptibility.

The study of coherent optical processes arouse increasing interest due to important applications of their novel quantum interference phenomena, such as electromagnetically-induced transparency (EIT)^1^ and absorption (EIA)^2^. EIT originates from destructive interference between different optical paths that causes the system to exhibit transparency for an incident pulse within a narrow spectral range with steep dispersion^3^. In contrast, EIA is a result of constructive interference due to transfer of coherence in Zeeman-degenerate systems^4^. Most recent research is concerned with multi-photon coherence processes^5^,^6^,^7^,^8^,^9^ and is carried out in multilevel atomic systems, including Lambda-, cascade- and N-type systems^10^,^11^,^12^.

Quantum dots (QD) are termed artificial atoms as they are confined in three dimensions and exhibit discrete energy levels^13^. The long coherence times, nonlinearity, and ease of integration exhibited by QDs makes them ideal candidates for quantum information processing^14^,^15^,^16^. QDs that are coupled through tunneling have rich energy levels and flexible methods of electronic/optical control further increasing the possible applications of QDs^17^,^18^. EIT and tunneling induced transparency (TIT) phenomena have been observed in CQDs^19^,^20^. The main difference between EIT and TIT is that EIT usually requires an external-field excitation and induced coherence, while TIT originates from tunneling induced coherence which is decided by the structure of the QDs. The interference mechanism^21^,^22^, pulse propagation^23^,^24^, and Kerr nonlinearity^25^,^26^,^27^ in TIT have been extensively studied for applications in solid state qubits and quantum electrodynamics^28^; however, few have studied the TIA and related nonlinear optical properties despite the potential applications of TIA in solid-state quantum devices and as a classical analog of EIA^29^ or its role in two photon-absorption^30^ and photon-assisted absorption^31^,^32^.

In this paper, we investigate the nonlinear TIA phenomenon and its physical mechanism. The resonant tunneling induces constructive interference for the nonlinear absorption whose peak value could be enhanced by more than ten times while its linewidth can be reduced to one tenth compared to the absorption without tunneling. Analytical expressions for the third and fifth-order nonlinearities are given in the weak-field limit, and the numerical result shows that contribution to the total absorption from the fifth-order nonlinear term dominates the third-order contribution. The competition of third- and fifth-order nonlinearities leads to anomalous dispersion in the total susceptibility. The value of the real part of the third-order nonlinearity changes from positive to negative along with variation of the tunneling detuning. Increased understanding and the ability for coherent control of the nonlinear processes in TIA may aid in future exploration of nonlinear tunneling dynamics and the design of novel absorption and refraction engineered devices.

## Results

### Model and basic equation

**Figure 1:**
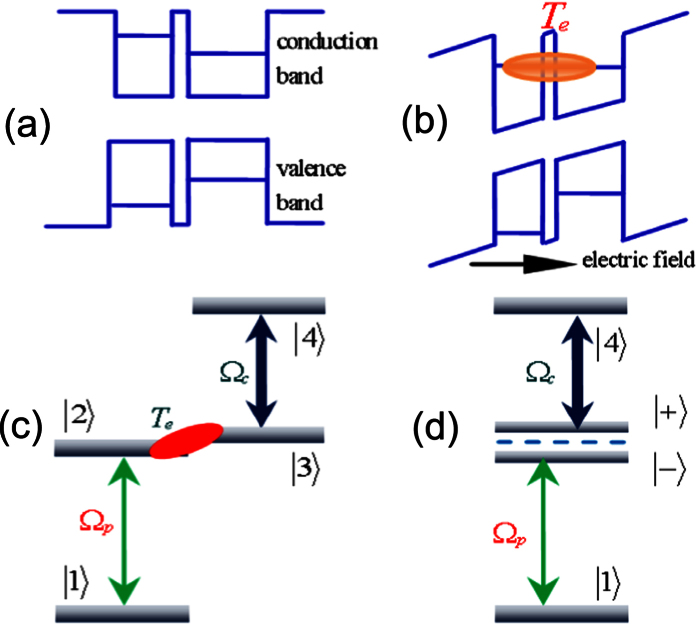
Schematic band structure of the QDM system (**a**) without and (**b**) with an applied electric field, (**c**) schematic level configuration and (**d**) corresponds to generic model (**c**) after diagonalizing the interaction with the tunneling Te, where two new eigenstates 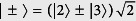.

We consider a quantum dot molecule (QDM) system where each QDM consists of two dots with different band structures coupled by tunneling^33^,^34^. A lateral device geometry appears more convenient for the application of bias voltages to QD ensembles with QDs of different sizes. A recent experiment reports self-assembled lateral QDMs can be produced on GaAs (001) substrates by a unique combination of molecular beam epitaxy and precise *in situ* atomic layer etching. This provides homogeneous ensembles of QDMs with a low density, where the two dots of the QDM are aligned along the 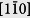 direction^35^. For a QDM, two dots are separated by a few nanometers of a barrier material. Schematic band structure and level configuration of an individual QDM system are shown in Fig. 1(a). We use Dirac bra-ket notion |e_L_, n_L_〉|e_R_, n_R_〉 to express the excitonic states, where the left (right) Dirac symbol gives the number of electrons and holes in the left (right) dots with subscript L (R). |1〉 = |0, 0〉|0, 0〉 without excitation is the ground state. A probe field can excite an electron from the valence band to the conduction band of a dot and a direct exciton forms, |2〉=|1, 1〉|0, 0〉. The excited electron can tunnel to the other dot and a indirect exciton appears, |3〉 = |0, 1〉|1, 0〉. A control field can further excite an electron in the conduction band forming a biexciton, |4〉 = |1, 2〉|1, 0〉^17^. Here we limit our discussion to the four QDM states above, as shown in Fig. 1(c). The application of the bias voltage causes the energy separation between the hole levels to become large so that the hole tunneling can be neglected (see Fig. 1(b))^36^. The cascade-type biexciton system has already been examined by pump-probe^17^ and two-photon absorption experments^30^ and proposed for studying entanglement dynamics^37^. Similar artificial structures have also been used for tunneling induced transparency and related applications^38^,^39^,^40^,^41^.

The Hamiltonian of the CQD system can be described in matrix form using the electric-dipole and the rotating-wave approximations,


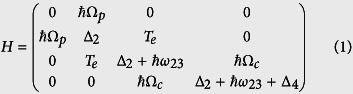


where the probe field detuning 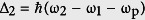, the control field detuning 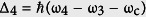 with the frequency of the probe (control) field ω_p_ (ω_c_). The tunneling detuning 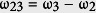, with the eigenfrequency of state |i〉 given by ω_i_. The tunneling matrix element T_e_ of an electron represents the magnitude of the coupling between the states |2〉 and |3〉. The parameters ω_23_ and T_e_ related to the interdot tunneling can be controlled by an electric field. The Rabi frequencies of the control and probe fields are 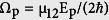 and 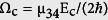, respectively, with the dipole momentum matrix element mi j from state|i〉 to state |j〉 and the laser electric field amplitude Ep, c. The system dynamics are described by Liouville-von Neumman-Lindblad equation:


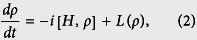


where ρ is the density matrix operator and 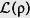 represents the Liouville operator that describes the decoherence process^20^. We obtain the following evolution of the differential equation for the density matrix element 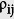:


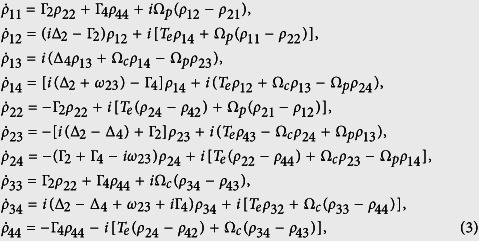


with 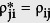, and the closure relationship 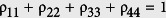. Here 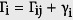 represents the effective decay rate, 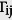 indicates spontaneous decay from |i〉 to |j〉 and 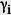 is the dephase term.

## Discussion

Here we consider a weak probe field and are interested in high-order nonlinearity of the CQDs. The effective susceptibility is given by^42^


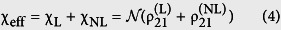


where the first term is the linear susceptibility, and the second term is the nonlinear susceptibility. By solving Eq. (3) in the weak-field limit with the iterative method, we obtain approximate solutions for the linear and nonlinear susceptibilities as follows:


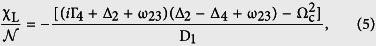



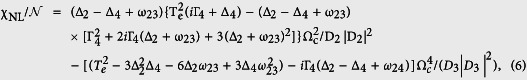


with


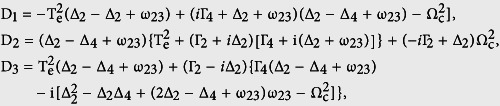


Where 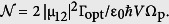 The optical confinement factor 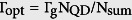
43 describes the fraction of the optical power guided in the QDs, where 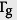 is the ratio of the volumn of all QDs and the mode volume, 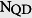 denotes the density of the QDs in active region and 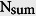 is the total density of the QDs as determined by experimental surface imaging. V is the effective mode volume of a single QD.

We may refer to realistic parameters for InAs self-assembled QDs^44^. For simplicity, the effective decay rate is assumed to be described by 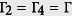 which are about 10 meV. If one takes N_QD_ = 0.6 × 10^10^ cm^−2^ and N_sum_ = 2 × 10^11^ cm^−2^, then 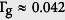 and 
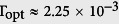
 are estimated following the procedure of ref. 43. In the model provided in ref. 45, a QD is treated as a disk with a diameter of 8 nm and a height of 10 nm; its effective mode volume is 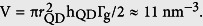


Here we focus on relative changes in the linear and nonlinear susceptibilities and the mechanism of competition between them, so the dimensionless quantities 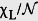 and 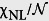 will be used in the following discussion for convenience.

**Figure 2:**
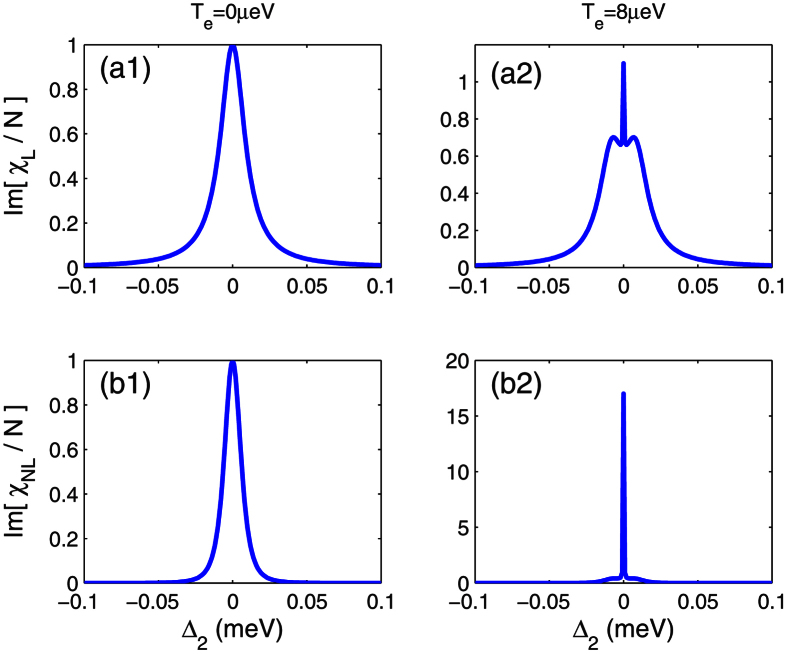
Linear absorption (top row) (**a1**) without tunneling T_e_ = 0 μeV and (**a2**) with tunneling T_e_ = 8 μeV; nonlinear absorption (bottom row) with (**b1**) T_e_ = 0 μeV and (**b2**) T_e_ = 8 μeV. Ω_c_ = 2 μeV, Δ_c_ = 0 μeV, and ω_23_ = 0 μeV.

Figure 2 shows the comparison of linear and nonlinear absorptions with and without the resonant tunneling, respectively. When the bias voltage is turned off, the interdot tunneling for two neighboring QDs is weak due to their different sizes and can, therefore, be neglected. There is a Lorentz-like absorption peak at a resonant frequency in both the linear and nonlinear susceptibilities, which means the QDMs behave like a two-level absorption medium (see Fig. 2(a1) and (b1)). When the bias voltage is applied, the individual QD electron levels come into resonance, and the resonant tunneling arises. The tunneling rate depends strongly on the barrier, which determines the decay rate of the electronic states, and responsible for their occupation. For convenience of calculation, the tunneling is usually scaled by the decay rate of a direct state. We focus on the weak coupling regime^20^ and interferences based TIT, so the value of Te is relatively small. If one takes T_e_ = 0.8 μeV^46^,^47^, there is an enhancement in linear and nonlinear responses (see Fig. 2(a2) and (b2)). It is interesting that the nonlinear absorption can be enhanced by a factor of nearly 18 under the given condition. The absorption linewidth is also drastically narrowed. The simulated full width at half maximum (FWHM) of 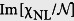 with Te = 0 is approximately 14 meV: larger than the exciton linewidth 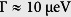. When the tunneling arises, the nonlinear absorption peak narrows considerably and its FWHM becomes approximately 1 μeV which is a sublinewidth level.

This can be understood in the dressed-state picture. The interdot tunneling couples the states |2〉 and |3〉 and leads to a pair of new eigenstates, 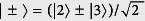
(see Fig. 1(d)). There are two optical pathes with the probe and control transitions, 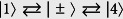. Constructive interference in absorption between two pathes occurs, and the nonlinear absorption builds up substantially. The resonant tunneling induces the inherent coherence of the system, and the inter-path quantum interference leads to enhanced absorption. This phenomenon is referred to as TIA.

**Figure 3:**
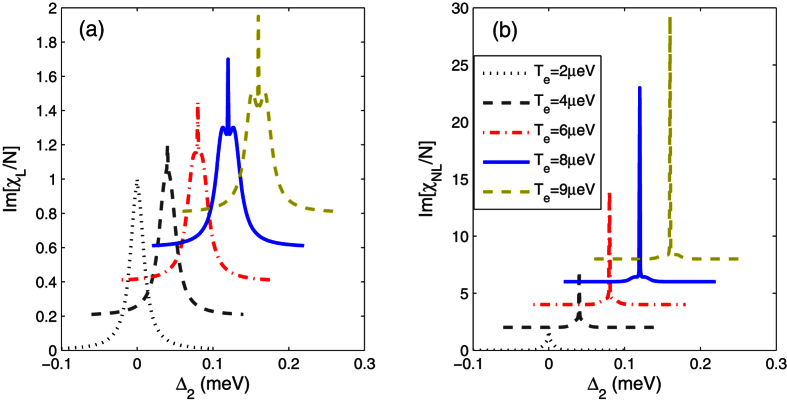
(**a**) Linear 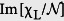 and (**b**) nonlinear absorptions 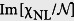 change with Te. Other parameters as Fig. 2(b2).

Figure 3 shows changes in the linear 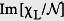 and nonlinear 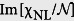 absorptions with tunneling T_e_. The linear absorption grows slowly as the tunneling increases, while the nonlinear absorption increases rapidly. Since the interdot tunneling occurs in the weak-coupling regime, the constructive interference between the two optical pathes plays an import role in the enhanced absorption.

**Figure 4:**
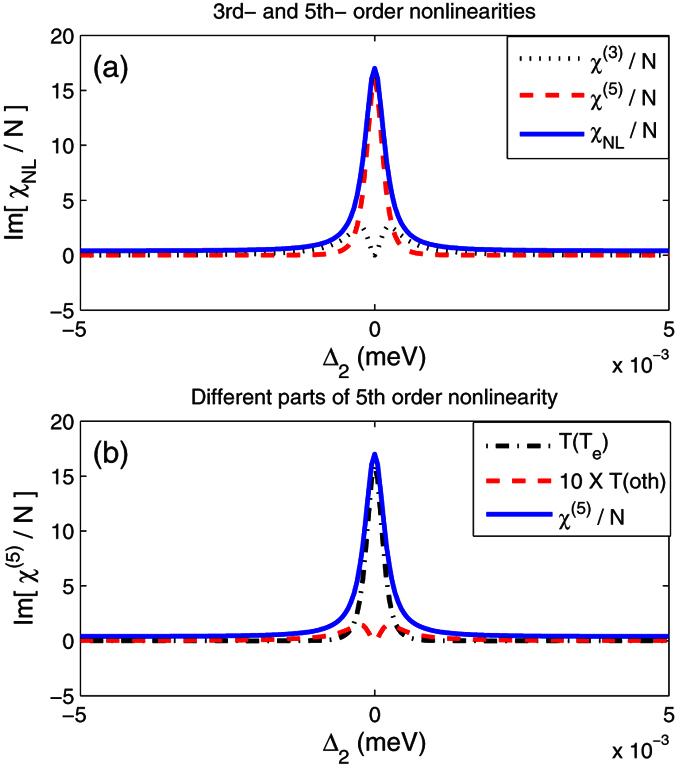
Comparison of (**a**) different nonlinear absorptions and (**b**) different parts of fifth-order nonlinearity under same conditions of Fig. 2(b2).

It is instructive to examine the enhanced nonlinear absorption. The nonlinear susceptibility 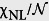 can be divided into two parts (see Eq. (6)). The first part is related to 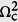 and belongs to a three-order nonlinear term 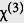; the other one involves 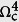 and is a fifth-order nonlinear term 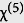. We compare 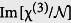, 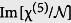 and the total absorption 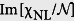 in Fig. 4(a). Figure 4 shows that 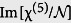 exhibits strong absorption at the resonance frequency and coincides well with 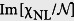. This means that the fifth-order nonlinear absorption is the primary contributor to the enhanced nonlinear absorption.

We also note the fifth-order nonlinearity contains the tunneling term denoted by 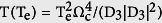 and other terms denoted 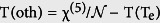. The two parts are compared in Fig. 4(b). The tunneling term 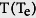 dominates the fifth-order nonlinearity, which also originates from tunneling-induced interference. So we conclude that it is the tunneling that leads to constructive interference for the fifth-order nonlinearity resulting in the enhanced nonlinear absorption.

**Figure 5:**
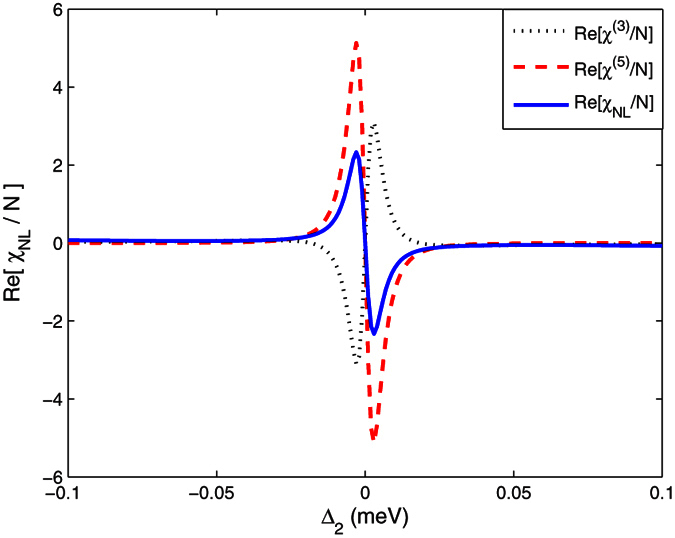
Comparison of third- and fifth-order nonlinear dispersions with same parameters as Fig. 2(b2).

The above discussion deals mainly with the nonlinear absorption. Next, we turn to properties of nonlinear dispersions. The third-order 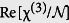, fifth-order 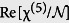 and total susceptibilities 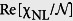 are compared in Fig. 5. 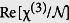 shows a normal dispersion at the resonant frequency, while 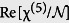 presents a strong anomalous dispersion. The competition between the third- and fifth-order susceptibilities make the total nonlinear susceptibility 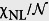 exhibit an anomalous dispersion.

**Figure 6:**
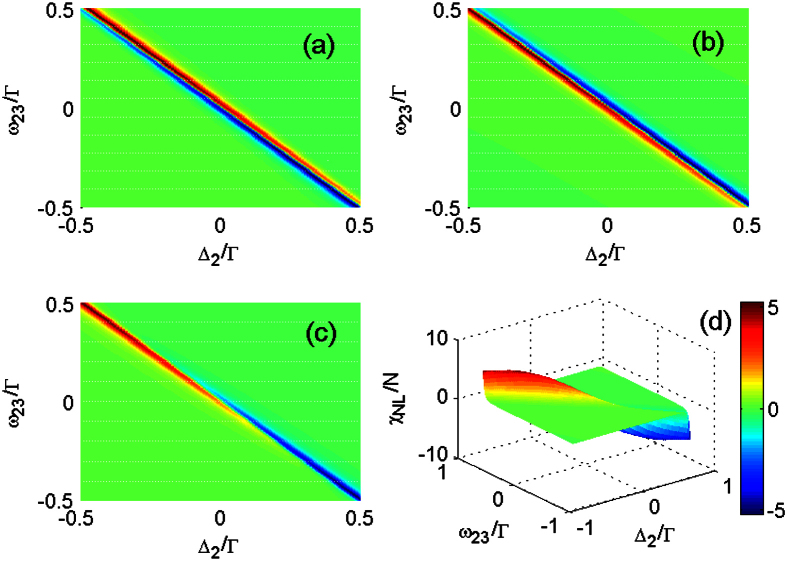
Comparison of the real parts of different nonlinear susceptibilities, (**a**) 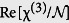, (**b**) 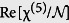 and (**c**) 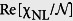 and the corresponding three dimensional view of the total susceptibility (**d**). Ω_c_ = 5 μeV, T_e_ = 20 μeV. All parameters are scaled by Γ.

Further detailed analyses of 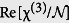, 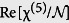 and 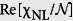 show 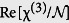 and 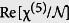 have opposite responses to changes in the tunneling detuning 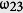 and laser detuning 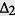, as shown in Fig. 6. A red (blue) color represents a positive (negative) value of the real part of the nonlinear susceptibility. First, we consider the case of ω_23_ = 0.5Γ and Δ_2_ = −0.5Γ (all parameters are scaled by Γ for simplicity). In Fig. 6(a), the region above the diagonal shows a high positive value and dominates 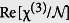, while the region below the diagonal part shows a small negative value. In contrast, in Fig. 6(b), the region above the diagonal indicates that 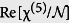 has a small negative value, but the region below the diagonal has a high positive value. Next, when ω_23_ decreases from 0.5Γ to −0.5Γ and Δ_2_ varies from −0.5Γ to 0.5Γ, both 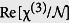 and 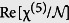 change greatly: the positive parts decrease in magnitude while the negative parts increase in magnitude. Finally, the competition between 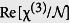 and 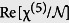 makes 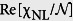 change from a positive value to a negative value when ω_23_ decreases from 0.5Γ to −0.5Γ and Δ_2_ increase form −0.5Γ to 0.5Γ, as shown in Fig. 6(c) and (d). The resonant tunneling induces the enhanced third- and fifth-order nonlinearities and their competition results in novel changes in the nonlinear absorption and dispersion.

In summary, we have investigated the nonlinear TIA process in a CQD system. Due to resonant tunneling, the nonlinear absorption increases dramatically. This is because the tunneling induces constructive interference in the nonlinear absorption. The simulated result shows that the nonlinear absorption could be enhanced by at least one order of magnitude. Further examination of the nonlinear susceptibility shows that it contains two parts, the third- and fifth-order terms, and it is the fifth-order nonlinear absorption that leads to the large total absorption. The competition of third- and fifth-order dispersions result in an anomalous dispersion in the total nonlinearity. Our work can aid in the understanding of high-order nonlinear processes and may be useful in designing absorption- and dispersion-engineered devices.

## Methods

All the simulated results were obtained by solving the density matrix equations in steady state with the electric-dipole and the rotating-wave approximation. The analytical expression is obtain by using the Mathematica, and the figures are plotted by using the Matlab.

## Additional Information

**How to cite this article**: Peng, Y. *et al*. Tunneling induced absorption with competing nonlinearities. *Sci. Rep.* **6**, 38251; doi: 10.1038/srep38251 (2016).

**Publisher's note:** Springer Nature remains neutral with regard to jurisdictional claims in published maps and institutional affiliations.
